# Serine protease inhibitor derived from *Trichinella spiralis* (TsSERP) inhibits neutrophil elastase and impairs human neutrophil functions

**DOI:** 10.3389/fcimb.2022.919835

**Published:** 2022-10-25

**Authors:** Porntida Kobpornchai, Onrapak Reamtong, Orawan Phuphisut, Preeyarat Malaitong, Poom Adisakwattana

**Affiliations:** ^1^ Department of Helminthology, Faculty of Tropical Medicine, Mahidol University, Bangkok, Thailand; ^2^ Department of Molecular Tropical Medicine and Genetics, Faculty of Tropical Medicine, Mahidol University, Bangkok, Thailand

**Keywords:** *Trichinella spiralis*, serine protease inhibitor, immunomodulatory molecules, neutrophil (PMN) functions, inflammatory disorders

## Abstract

During early infection with *Trichinella spiralis*, host neutrophils destroy newborn larvae migrating in the bloodstream, preventing infection. However, parasites secrete various immunomodulatory molecules to escape the host’s defense mechanisms, allowing them to infect the host and live for long periods. *T. spiralis* secretes serine protease inhibitors (TsSERPs), which are key inhibitory molecules that regulate serine proteases involved in digestion and inflammation. However, the modulatory roles of TsSERP in the inhibition of neutrophil serine proteases (NSPs) and neutrophil functions are unknown. Therefore, the immunomodulatory properties of recombinant TsSERP1 (rTsSERP1) on NSPs and neutrophil functions were investigated in this study. rTsSERP1 preferentially inhibited human neutrophil elastase (hNE). In addition, incubation of rTsSERP1 with fMLP-induced neutrophils impaired their phagocytic ability. The formation of neutrophil extracellular traps (NETs) was activated with phorbol myristate acetate (PMA), and NETs were dramatically reduced when treated with rTsSERP1. Furthermore, rTsSERP1 suppressed the production of proinflammatory cytokines and chemokines during neutrophil activation, which are essential for neutrophil-mediated local or systemic inflammation regulation. In conclusion, *T. spiralis* immune evasion mechanisms are promoted by the inhibitory properties of TsSERP1 against neutrophil elastase and neutrophil defense functions, and these might be promising alternative treatment targets for inflammatory disorders.

## Introduction

*Trichinella spiralis* is a harmful food-borne zoonotic nematode that has a detrimental effect on human and animal health worldwide (Murrell and Pozio, 2011). Infection occurs after ingesting raw or inadequately cooked meat carrying encapsulated first-stage larvae (TsL1). The parasite matures to the adult stage in the small intestine and releases newborn larvae (NBL), which infect muscle tissues and remain there as the infective stage (TsL1) for several years (Fröscher et al., 1988). To survive long-term in their hosts, *T. spiralis* uses various methods to modulate host defenses, including intracellular infection, nurse cell formation, and immunomodulation (Despommier, 1998; Ashour, 2013). Previous studies reported all life stages of *T. spiralis* released immunomodulatory molecules that modulated host cellular and humoral immune responses, which altered immune cells such as macrophages, dendritic cells, neutrophils, and lymphocytes (Aranzamendi et al., 2012; Bai et al., 2012; Song et al., 2019; Sun et al., 2019).

Serine protease inhibitors or serpins (TsSERPs) have been identified in excretory-secretory products of all developmental stages of *T. spiralis*. These proteins not only play important roles in larval infectivity (Song et al., 2018; Yi et al., 2020), they also regulate host anti-inflammatory immune responses by mediating alternatively activated macrophages (AAMϕ), regulatory T cells (Treg), CD8^+^CD28^−^ immunosenescent T cells, and regulatory B cells (Breg) (Xu et al., 2019; Xu et al., 2021b). Neutrophils are key cells that have critical roles in limiting *T. spiralis* infection (Bass and Szejda, 1979a; Bass and Szejda, 1979b). However, *T. spiralis* can evade neutrophil immunity and cause chronic infection. How *T. spiralis* evades neutrophil immune mechanisms is unknown.

During neutrophil immune responses, the release of neutrophil serine proteases (NSPs) including neutrophil elastase (NE), cathepsin G (CG), and proteinase-3 (PR3) facilitate direct killing by cleavage of pathogen outer membrane, phagocytosis, release of proinflammatory cytokines and chemokines, inflammation, and the formation of neutrophil extracellular traps (NETosis) (Pham, 2008; Cassatella et al., 2019). The NE has been associated with the formation of NETs. Mechanistically, the mitogen phorbol 12-myristate 13-acetate (PMA), an *in vitro* experiment inducer, activates the Raf-Mek-Erk pathway, which activates protein kinase C (PKC) and then produces ROS *via* NADPH oxidase, releasing NE from granules into the cytoplasm. The NE then translocates to the nucleus and cleaves histones, causing nuclear membrane disintegration, chromatin expansion, and finally cell lysis and NET release (Rosazza et al., 2021). Therefore, the inhibition of NSPs activity most likely necessitates *T. spiralis* escaping these frontline immune defenses. In a previous study, human serpins α1-PI, serpinB1, and secretory leukocyte inhibitor (SLPI) blocked NSPs, particularly NE, which reduced inflammation-related severity and excessive elastase-induced tissue damage in a mouse model of chronic obstructive pulmonary disease (COPD) (Kelly-Robinson et al., 2021).

Several TsSERP isoforms have been identified and characterized. The first TsSERP isoform (TsSERP1) identified by (Nagano et al., 2001) was studied further because it selectively inhibited neutrophil elastase (found in this study). Subsequently, the inhibitory properties of TsSERP1 on neutrophil functions including phagocytosis, NETosis, and cytokine and chemokine responses were described. The impact of TsSERP1 on neutrophil functions described here will help researchers better understand *T. spiralis* immunology, and the underlying immunomodulatory mechanisms might be an alternative therapeutic target for inflammatory diseases.

## Materials and methods

### Ethics statement

All human study and data experiment protocols were reviewed and approved by the local Institutional Review Board, The Human Research Ethics Committee of the Faculty of Tropical Medicine, Mahidol University, Bangkok, Thailand (MUTM 2021-013-01), with all participants signing informed consent prior to blood collection. All experiments were performed in accordance with relevant guidelines and regulations.

### Protease inhibitory assay

rTsSERP1 (accession no. AF231948) was expressed in *Escherichia coli* strain M15 and purified by CO^2+^ affinity chromatography (Clontech Laboratories, Inc., Mountain View, CA) according to our previous study (Nuamtanong et al., 2019) (**Supplementary Figure 1**). The inhibitory activity of rTsSERP1 against human neutrophil serine proteases (hNSPs) including neutrophil elastase (hNE) (Fitzgerald Industries International, Acton, MA), proteinase 3 (hPR3) (Diarect AG, Freiburg, Germany), and cathepsin G (hCG) (Calbiochem, Darmstadt, Germany) were evaluated by hydrolysis of specific substrates, fluorogenic (FRET) or colorimetric substrates, according to previous publications with some modifications (Korkmaz et al., 2008; Tausch et al., 2009; Perera et al., 2013; Anderson et al., 2019). Briefly, each neutrophil protease was mixed with rTsSERP1 at molar ratios (enzyme: inhibitor) of 1:0, 1:1, 1:2, 1:4, and 1:10 and then incubated at 37°C for 30 min. The fluorogenic substrates of hNE (MeOSuc-AAPV-AMC; Calbiochem) or hPR3 (Abz-VAD-norV-ADRQ-EDDnp; Alta Biosciences, Birmingham. UK), or colorimetric substrates of hCG (Suc-AAPF-pNA, Calbiochem) were added and then the enzymatic activities were monitored using a Synergy™ H1 Hybrid Multi-Mode Reader (BioTek, Winooski, VT, USA). An alpha-1 proteinase inhibitor (α1PI) (Athens Research & Technology, Inc., Athens, GA) was used as a positive control for both assays. All experiments were performed in triplicate.

The inhibition constant (K_i_) of TsSERP1 for hNE was determined using a Dixon plot according to a previous publication with some modifications (Toubarro et al., 2013). rTsSERP1 (0, 20, 40 and 80 nM) was incubated with 10 nM of hNE for 15 min at 37°C; then, 50, 100 and 200 µM of MeOSuc-AAPV-AMC (Calbiochem) was added to the elastase reactions.

### Isolation of human neutrophils

Human neutrophils were isolated from peripheral venous blood of three healthy donors aged between 20-50 years old according to a previous publication with some modifications (Heit et al., 2006). In summary, 20 ml peripheral venous blood was drawn from each donor followed by collection in heparinized tubes to avoid blood clotting. The red blood cells (RBCs) were removed using dextran sedimentation. Subsequently, the leukocyte-rich plasma separated from the top layer was transferred into a 15 ml conical tube followed by centrifugation at 700 ×g for 10 min and the supernatant was discarded. The remaining RBCs were lysed with ice-cold ddH_2_O for 30 s and then mixed with 0.6 M KCl prior to dilution with 1×PBS before centrifugation. The pellet was resuspended in 1×PBS and the polymorphonuclear cells (PMNs) were collected by gradient centrifugation using lymphocyte separating medium (LSM; MP biomedicals, Cat. No:50494, Illkirch, France). The viability and purity of neutrophils were determined using trypan blue and Giemsa staining, respectively, which revealed that they were > 90% viable and 95% neutrophils pure, but with only 2% eosinophil, 1% monocyte and 2% lymphocytes contamination.

### Cytotoxicity analysis of rTsSERP1

The cytotoxicity of rTsSERP1 on human neutrophils was evaluated by MTT assay kit (Sigma-Aldrich, Cat. NO:11465007001, Saint Louis, MO, USA) according to manufacturer’s protocol. The cells were seed into a 96-well culture plate at a density of 3 × 10^4^ cells/200 µl/well, followed by treatment with different concentrations of rTsSERP1 (0, 1.25, 2.5, 5, 10, and 20 µg/ml). Media without the cells was used as the blank. After incubation for 4 h, the 0.5 mg/ml of MTT solution was added, followed by additional incubating for 4 h. The supernatant was discarded prior to add 100 µl of DMSO. Subsequently, the optical density (OD) at a wavelength of 570 nm was measured using the Sunrise™ absorbance reader (Tecan Group Ltd, Männedorf, Switzerland). All assays were performed in triplicate with three donors and the percentage of cell viability was calculated as below.


$$\text{\% cell viability}=\frac{\text{OD of treatment group }-\text{ OD of blank control}}{\text{OD of untreated group }-\text{ OD of blank control}}\times 100$$

#### Inhibitory effect of rTsSERP1 on neutrophil elastase released from human neutrophils

Elastase release and inhibition assay were performed according to previous publication with some modifications (Morais et al., 2018). Briefly, human neutrophils were plated into a 96-well culture plate at a density of 2 × 10^5^ cells/200 μl/well. Elastase release and inhibitory effects were induced by stimulation with 100 nM of N-formylmethionyl-leucyl-phenylalanine (fMLP) (dissolve in DMSO) (Sigma-Aldrich, Cat. No:F3506, Saint Louis, MO, USA) for 10 min prior to the addition of 40 μM of rTsSERP1 or recombinant mouse dihydrofolate reductase (rmDHFR) (irrelevant control protein) at 37°C for 1 h. Treatment with fMLP alone and non-treatment (only media) were used as positive and negative controls, respectively. After incubation, 100 ng/ml of colorimetric neutrophil elastase substrate (elastase substrate I; Calbiochem^®^, Cat. No:70967-90-7) was added into each reaction and the colorimetric reaction was immediately monitored at a wavelength of 405 nm every 10 min until 1 h. The percentage of neutrophil elastase activity was calculated at 1 h of kinetic assay. The experiment was performed in triplicate and the results were reported as a change in absorbance per minute. 4-(2-aminoethyl) benzenesulfonyl fluoride hydrochloride (AEBSF) (Sigma-Aldrich, Cat. No:A8456, Saint Louis, MO, USA) at 0.1 mM was used as an inhibitory control for neutrophil elastase. All assays were performed in triplicate with three donors.

#### Inhibitory effect of rTsSERP1 on neutrophil phagocytosis

The effect of rTsSERP1 on neutrophil phagocytic functions was evaluated by a Vybrant™ Phagocytosis Assay Kit (Thermo Fisher Scientific Inc., Cat No: V6694, Waltham, USA) according to the manufacturer’s protocol. Human neutrophils at a density of 2 × 10^5^ cells/well were placed into each well of a 96-well fluorescent microplate. The experiment and control were set up under four different conditions including 1) only fluorescent *E. coli* BioParticle as a blank control, 2) untreated neutrophils (only media) incubated with fluorescent *E. coli* BioParticle as mock, 3) fMLP + neutrophils incubated with fluorescent *E. coli* BioParticle as a positive control, and 4) fMLP + neutrophils treated with rTsSERP1 or rmDHFR and incubated with fluorescent *E. coli* BioParticle as the test sample. After cell adherence, neutrophils were stimulated by incubation with 100 nM fMLP for 30 min followed by adding 10 µg/ml rTsSERP1 or 10 µg/ml rmDHFR for 1 h. After removing the culture media, 100 µl of the fluorescent *E. coli* BioParticle suspension was added into each well and incubated further for 2 h. The BioParticle suspension was removed and added 100 µl of trypan blue to quench the fluorescence of the extracellular particles. The ability of phagocytosis was detected after removal of the trypan blue. The intracellular fluorescent signal of the engulfed particles was monitored using a fluorescence plate reader at 480 nm excitation and 520 nm emission. The phagocytotic response to the effector rTsSERP1 was calculated as follows:


$$\%\text{Effect}=\frac{\text{Mock or test sample}-\text{Blank control}}{\text{Positive control}-\text{Blank control}}\times 100\%$$

To determine the fluorescent phagocytotic effect, neutrophils were plated on a 24-well plate with a poly-L-lysine coated 12-mm glass coverslip. The cells were treated as for the above experiment. After incubation with the fluorescent *E. coli* BioParticle, the fresh cells were gently washed with 1×PBS three times, followed by nuclei counterstaining with Hoechst 33342 (Invitrogen, Cat No, H3570, California, USA) for 20 min. The cover slips were washed several times with 1×PBS and then mounted on a glass slide with FluorSave™ reagent (Calbiochem, Cat No. 345789, Darmstadt, Germany). Cells were analyzed using a laser scanning confocal microscope (LSM700, Zeiss, Jena, Germany). Digital images were captured using the Zeiss microscope software package ZEN 2012 (Zeiss, Jena, Germany).

#### Inhibitory effect of rTsSERP1 on neutrophil extracellular trap (NETosis)

The formation of NET was performed according to previous publication with some modifications (Barrientos et al., 2013). Neutrophils were prepared at a density of 1 × 10^6^ cells/ml in a 1.5 ml microcentrifuge tube. The Sytox green™ (Thermo Fisher Scientific Inc., Cat No:S7020, Waltham, USA), a DNA binding dye of impermeable cells, at a final concentration of 5 µM was added into the tube. Subsequently, 200 µl of resuspended cells were seeded into each well of a 96-well black polystyrene plate (Corning Incorporated, Corning, NY). After cell attachment, the cells were cultured with 10 µg/ml rTsSERP1 or 10 µg/ml rmDHFR (irrelevant control protein) and then incubated for 30 min at 37°C with 5% CO2. Neutrophil extracellular trapping (Anderson et al., 2019) was stimulated by adding 50 nM PMA (Sigma-Aldrich, Cat. No:P8139, Saint Louis, MO, USA) (dissolved in DMSO) and incubation for 4 h at 37°C with 5% CO2. Fluorescence was measured at excitation/emission wavelengths of 485/527 nm. Non-treatment (only media) was used as a negative control. All assays were performed in triplicate with three donors.

For fluorescent image analysis, the neutrophils from NET induction as above experiment at a density of 2.5 × 10^5^ cells were placed on each well of a 24-well plate containing poly-L-lysine coated 12-mm glass coverslips. The fresh cells were gently washed with 1×PBS three times. The coverslips at the bottom of the well were collected prior to being carefully placed on a glass slide containing a drop of FluorSave™ mounting reagent. Images were analyzed by observing staining patterns with a ×100 oil objective lens on a laser scanning confocal microscope (LSM700, Zeiss, Jena, Germany). Digital images were captured using the microscope software package ZEN 2012 (Zeiss). Non-treatment was used as a negative control.

#### Inhibitory effect of rTsSERP1 on cytokines and chemokines released from human neutrophils

The effect of rTsSERP1 on the inhibition of cytokines released from human neutrophils was determined by real-time RT-PCR (qRT-PCR) and ELISA. Human neutrophils were seeded into a 24-well culture plate at a density of 5 × 10^5^ cells/well followed by treatment with 10 µg/ml rTsSERP1 or 10 µg/ml rmDHFR (irrelevant control protein). After incubation at 37°C for 1 h, cells were stimulated with 100 nM fMLP and incubated for 4 h. Cells treated with fMLP alone and non-treated (only media) were used as positive and negative controls, respectively. After incubation, the cells and supernatants were collected to monitor the cytokine responses using qRT-PCR and ELISA, respectively. Treatment with 0.1 mM AEBSF was used as a positive inhibitory control. All assays were performed in triplicate with three donors.

### Real–time RT-PCR

Total RNA was isolated from neutrophils using TRIZOL reagent (Invitrogen) according to the manufacturer’s instructions. Genomic DNA contamination was removed by treating total RNA with DNaseI (Thermo Fisher Scientific) prior to conversion to cDNA using a RevertAid First-strand cDNA Synthesis Kit (Thermo Fisher Scientific, Cat. No: K1622, Waltham, MA). SYBR green qRT-PCR (BioRad, California, USA) was performed as mentioned elsewhere with some modifications (Kobpornchai et al., 2020). The reactions were amplified using specific primers for human cytokines and chemokines including IL-1β, IFN-γ, TNF-α, IL-6, IL-8 and CCL3 (**Supplementary Data 1**: **Table S1**). GAPDH was used as housekeeping gene. The amplification was performed with Eppendorf Realtime PCR (Realplex4, Eppendorf, Hamburg, Germany), consisted of a first denaturation step at 95°C for 5 min, followed by 40 cycles at 95°C for 15 s and 60°C for 60 s, and a melting curve step at 65–95°C. Relative mRNA expression was calculated using the comparative Ct method with the formula 2^-ΔΔCt^.

### Cytokine ELISA

Supernatants collected from the experiment above were obtained to determine the levels of IL-1β and IFN-γ cytokines using commercially available sandwich ELISA kits according to the manufacturer’s instructions (BioLegend, Cat. No;437004 (IL-1β), 430104 (IFN-γ), San Diego, CA). The cytokine concentrations were performed in triplicate and standard curves were generated with recombinant cytokines.

### Statistical analysis

Statistical analyses were performed with GraphPad Prism 6 software (GraphPad Software Inc., La Jolla, CA, USA). Significance of the differences between groups was analyzed using the one-way ANNOVA test. Individual data and mean ± S.D. of the group are presented. *p* value< 0.05 was considered significant.

## Results

### rTsSERP1 preferentially inhibits neutrophil elastase among NSP

**Figure 1:**
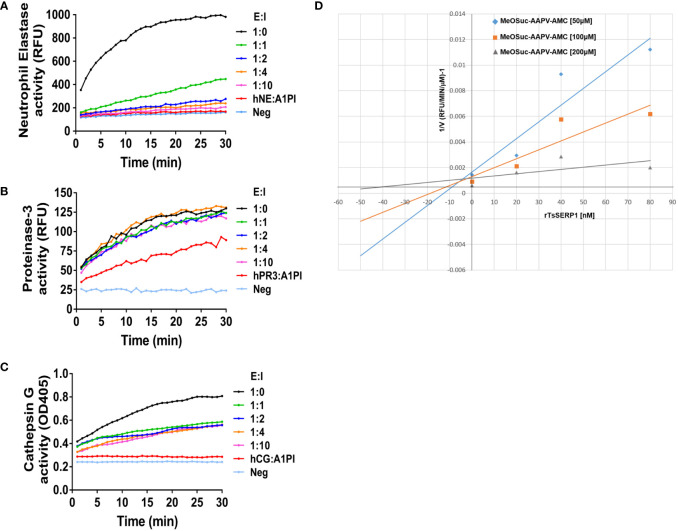
The inhibitory activity of rTsSERP1 against human neutrophil serine proteases (hNSPs). The inhibition of rTsSERP1 against NSPs including human neutrophil elastase (hNE) **(A)**, human proteinase 3 (hPR3) **(B)**, or human cathepsin G (hCG) **(C)** was assessed by enzyme inhibitory assays using specific peptide substrates. Each enzyme and rTsSERP1 was incubated at the molar ratios (enzyme: inhibitor) of 1:0, 1:1, 1:2, 1:4 and 1:10. Alpha-1 proteinase inhibitor (α1PI) was used as a positive control. The Dixon plot showed the inhibition constant (K_i_) of TsSERP1 for hNE **(D)**. The K_i_ was calculated from the inhibition of hNE by rTsSERP1 (0, 20, 40 and 80 nM) against the fluorogenic MeOSuc-AAPV-AMC substrate at three different concentrations (50, 100 and 200 µM).

Serpin forms an irreversible covalent complex with its specific protease to inhibit it, resulting in its inactivation and clearance from the circulation (Sanrattana et al., 2019). The inhibitory assay, which used specific peptide substrates, confirmed that rTsSERP1 completely inhibited hNE activity when the molar ratio (E:I) was increased to 1:2 (**Figure 1A**) and K_i_ value of approximately 5 nM was determined against hNE (**Figure 1D**). While rTsSERP1 only inhibited hCG partially even it did increase the molar ratio (E:I) to 1:10 (**Figure 1C**). There was no inhibitory effect of rTsSERP1 on hPR3 at all ratios (**Figure 1B**). The 3D structure of TsSERP1 was simulated using conserpin as a template. The molecular docking of TsSERP1 with NE showed that the reactive central loop (RCL) of serpin containing the inhibitory site of target protease specifically interacted with the structure of NE (**Supplementary Figure 2**).

**Figure 2:**
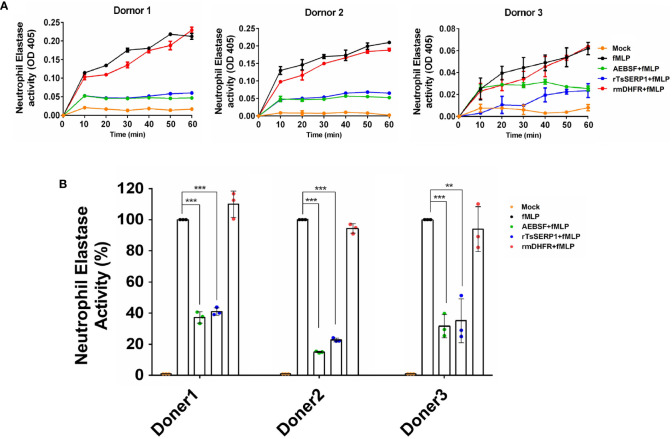
The neutrophil-secreted elastase activity inhibited by rTsSERP1. Neutrophils were induced with 100 nM of fMLP prior to treatment with 40 µM of rTsSERP1or an irrelevant control (rmDHFR) followed by detecting NE activity using a neutrophil elastase substrate for 1 h **(A)** and the percentage of the NE activity was calculated at 1h by setting neutrophils treated with fMLP alone as 100% activity **(B)**. Media alone was used as a negative control and 0.1 mM of 4-(2-aminoethyl) benzenesulfonyl fluoride hydrochloride (AEBSF) was used as a positive control for serine protease inhibition. The bar charts show the data of three different donors, which are presented as the mean ± SD. The experiments were performed in triplicate with three independent experiments. One-way ANOVA followed by a Bonferroni multiple comparison test were used for statistical analysis: **p < 0.01 and ***p < 0.001. The treatment conditions were included media alone (Mock), only fMLP induction (fMLP), fMLP induction and AEBSF treatment (AEBSF+fMLP), fMLP induction and rTsSERP1 treatment (rTsSERP1+fMLP), fMLP induction and irrelevant control (rmDHFR) treatment (rmDHFR+fMLP).

In addition, the granule content produced by human neutrophils was used to test the inhibitory effect of rTsSERP1 on hNE activity. The findings revealed that the neutrophil granule content released by fMLP activation strongly hydrolyzed the preferential NE substrate, which was set to 100% hNE activity. Pre-treatment of the granule content with rTsSERP1 prior to incubation with the substrate resulted in a reduction of hNE activity of 60%–80% in all three donors, which was comparable to treatment with AEBSF (a positive inhibitor control). Pre-treatment of the granule content with rmDHFR had no inhibitory effect on hNE activity. No hNE activity was observed when neutrophils were incubated with media alone (**Figure 2**). The aforementioned findings indicated that rTsSERP1 is a NE preference inhibitor. Treatment of neutrophils with DMSO alone at the same amount as that used to dissolve fMLP did not increase hNE activity (**Supplementary Figure 6A**).

### rTsSERP1 impaired neutrophil phagocytosis

**Figure 3:**
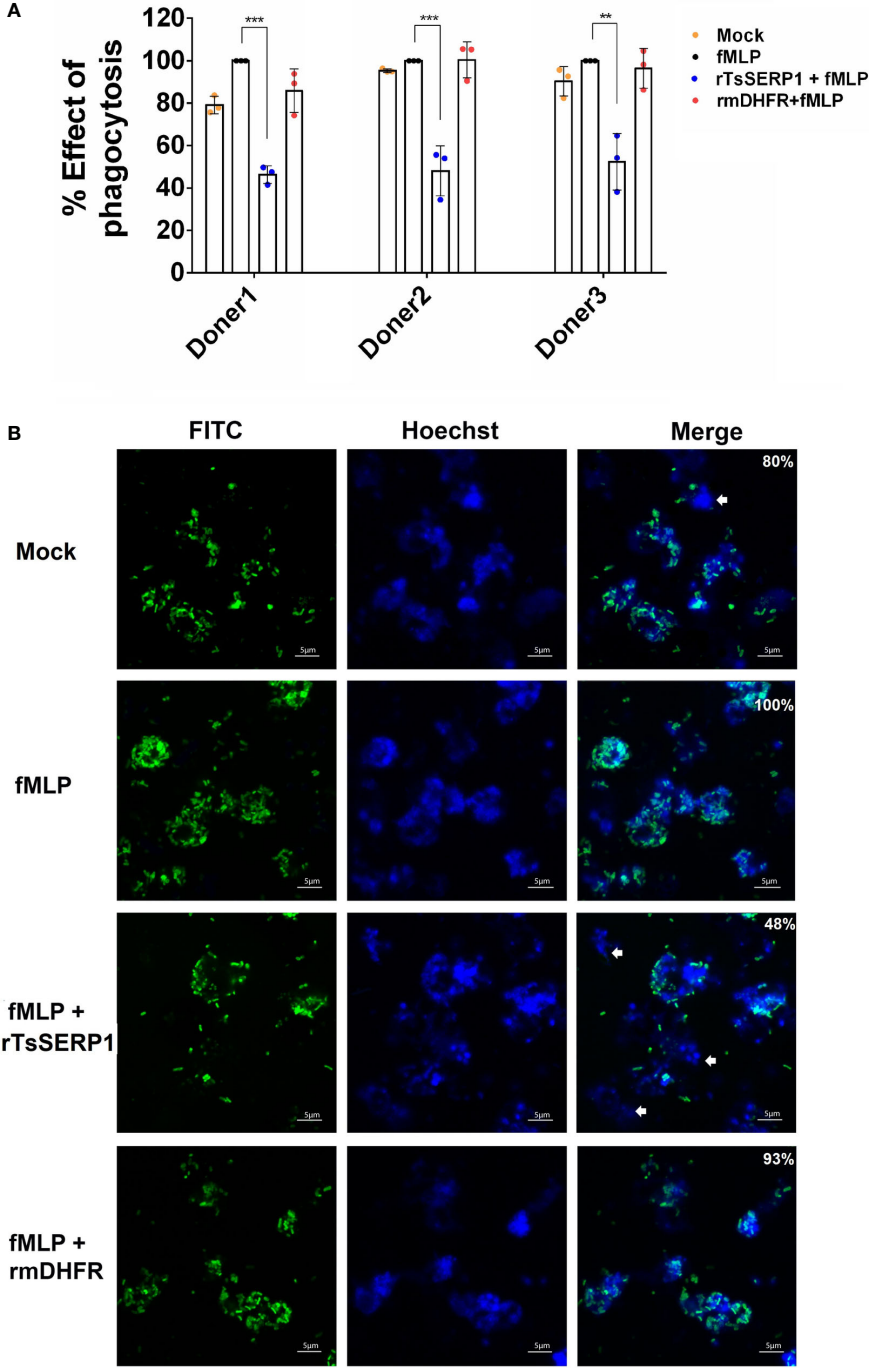
rTsSERP1 impairs neutrophil phagocytosis. The phagocytosis of neutrophils was induced by 100 nM of fMLP prior to treatment with 10 µg/ml of rTsSERP1 or an irrelevant control (rmDHFR), followed by incubation with the fluorescent *E. coli* BioParticle. After incubation, the fluorescent signal was measured and subsequently calculated as the percentage of phagocytosis **(A)**. Neutrophils treated with fMLP alone were set as 100% phagocytotic effect. The bar charts show the data of three different donors, which are presented as the mean ± SD. The experiments were performed in triplicate with three independent experiments. One-way ANOVA followed by a Bonferroni multiple comparison test were used for statistical analysis: **p < 0.01 and ***p < 0.001. Fluorescent images **(B)** were captured to confirm the above result. The percentage of phagocytosis positive out of the total number of alive neutrophils was indicated in the merge of picture. The *E. coli* BioParticle was labeled with FITC (green) and the nucleus was counterstained with Hoechst 33342 (blue). Examples of non-phagocytotic neutrophils are indicated by white arrows. The treatment conditions were included media alone (Mock), only fMLP induction (fMLP), fMLP induction and rTsSERP1 treatment (rTsSERP1+fMLP), fMLP induction and irrelevant control (rmDHFR) treatment (rmDHFR+fMLP).

Prior to evaluating the effect of rTsSERP1 on neutrophil functions, the optimal dose of rTsSERP1 for treating neutrophils was determined using a cell viability assay (MTT assay). rTsSERP1 at a concentration range of 1.25–20 μg/ml did not kill neutrophils (**Supplementary Figure 3**). Cells incubated with media alone were used as a negative control. All concentrations of rTsSERP1 were nontoxic and a concentration at 10 μg/ml was selected for the subsequent experiments. The impact of rTsSERP1 on neutrophil phagocytosis was analyzed using the fluorescent *E. coli* BioParticle. The treatment of fMLP-neutrophils with rTsSERP1 significantly reduced the neutrophil phagocytic activity as assessed by a 40%–50% reduction of the fluorescent signal compared with neutrophils induced with fMLP alone (the positive control was set to 100%) (**Figure 3A**). Treatment of fMLP-induced neutrophils with rmDHFR did not impair their phagocytotic activity (**Figure 3A**). Neutrophil phagocytosis was not increased when neutrophils were treated with DMSO alone at the same concentration as that used to dissolve fMLP (**Supplementary Figure 6B**).

Images of fluorescent bacterial phagocytosis by neutrophils were consistent with the fluorescent signal mentioned above, which revealed a decrease in the number of bacteria inside fMLP-induced neutrophils when treated with rTsSERP1 but not with rmDHFR (**Figure 3B**). Z-stack analysis confirmed bacteria were internalized by neutrophils (**Supplementary Figure 4**). The same results were obtained for all three donors. These findings imply that rTsSERP1 interferes with neutrophil phagocytic activity.

### rTsSERP1 reduced NETosis

NETosis, the process of neutrophil cell death whereby neutrophil extracellular traps (NETs) capture and kill bacteria and other pathogens, is thought to have a key role in the entrapment and eradication of microbes (Majewski et al., 2016). In previous studies, NE inhibitors such as small molecule inhibitors, SLPI, and serpin B1 regulated and decreased NET production (Majewski et al., 2016; Kasperkiewicz et al., 2020). During *T. spiralis* infection, neutrophils have an important role in frontline defense against larva invasion and migration (Bass and Szejda, 1979b) and NETs may contribute to larva entrapment as seen in *Haemonchus contortus* (Muñoz-Caro et al., 2015).

**Figure 4:**
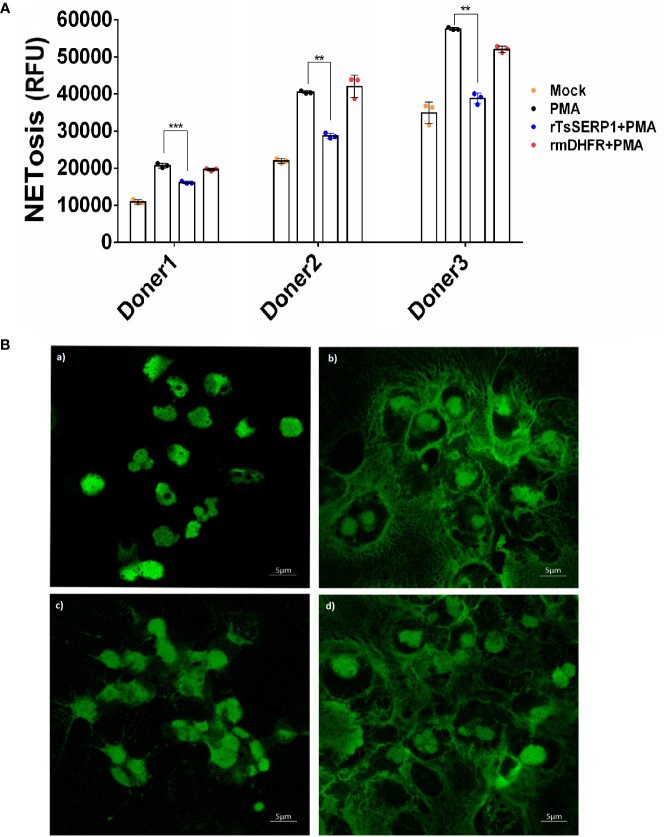
rTsSERP1 inhibits neutrophil extracellular traps (NETosis). Neutrophils initially stained with Sytox green (5 µM) were treated with rTsSERP1 or rmDHFR (10 µg/ml) for 30 min followed by induction with PMA (50 nM) for 4 h. NETosis was analyzed by the quantitative fluorescence intensity **(A)** and fluorescent images **(B)** with different treatment conditions including media alone (Mock) (a), PMA induction alone (PMA) (b), rTsSERP1 treatment before PMA induction (rTsSERP1+PMA) (c), and treatment with an irrelevant control (rmDHFR) before PMA induction (rmDHFR+PMA) (d). The bar charts show the data of three different donors, which are presented as the mean ± SD. The experiments were performed in triplicate with three independent experiments. One-way ANOVA followed by a Bonferroni multiple comparison test were used for statistical analysis: **p < 0.01 and ***p < 0.001.

To investigate whether the effect of TsSERP1 limited the NETosis of neutrophils, the fluorescent level of Sytox green-stained DNA released from cells and nuclear DNA of dead cells was quantitatively analyzed. The results demonstrated that fluorescent signals were markedly increased in PMA-induced neutrophils from all three donors when compared with untreated controls. Surprisingly, rTsSERP1 treatment of PMA-induced neutrophils prevented NETosis, which resulted in a significant reduction in fluorescent targeted extracellular DNA (doner 1; *p*<0.001 and donors 2 and 3; *p*<0.01). Treatment with rmDHFR (an irrelevant control) had no effect on NETosis (**Figure 4A**). The fluorescent images were also used to confirm the fluorescent signal described above and both results were found to be in concordance (**Figure 4B**). Neutrophils did not form NETs in the absence of PMA stimulation or treatment with rTsSERP1 or rmDHFR alone (**Supplementary Figure 5**). The same amount of DMSO used to dissolve PMA did not also promote the production of NETs when applied to neutrophils (**Supplementary Figure 6C**). From the findings of this study, we can conclude that TsSERP1 may decrease NETosis, which could be attributable to NE inhibition.

### rTsSERP suppressed neutrophil cytokine and chemokine responses

Inhibiting NE reduced the amount of pro-inflammatory cytokines and chemokines secreted by neutrophils, lowering host resistance to *Pseudomonas aeruginosa* (Benabid et al., 2012a). TsSERP isoform (*Ts*-serpin), which was discovered in TsL1’s ES product, inhibited NE and demonstrated an anti-inflammatory response by alternative activation of macrophages (Xu et al., 2021b). However, no research has been done on the regulatory function of TsSERP on neutrophil inflammation.

**Figure 5:**
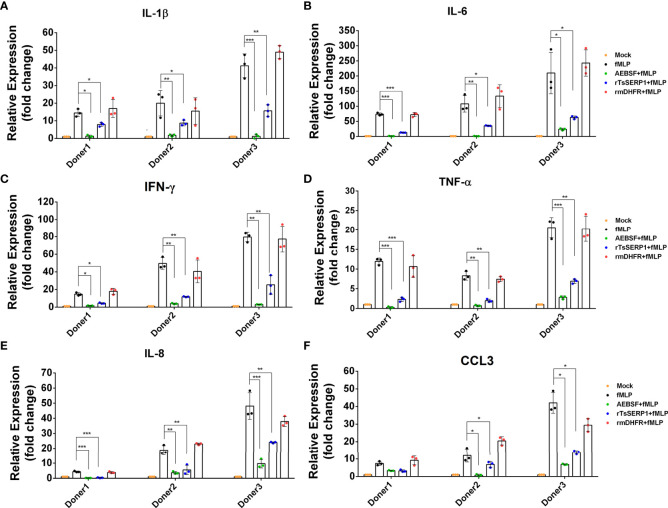
rTsSERP1 suppresses the transcription levels of cytokine and chemokine mRNAs in human neutrophils. Neutrophils were treated with 10 µg/ml of rTsSERP1 or an irrelevant control (rmDHFR) for 1 h prior and then stimulated with 100 nM of fMLP for 4 h. Thereafter, the cells were collected for RNA isolation and qRT-PCR analysis for IL-1β **(A)**, IL-6 **(B)**, IFN-γ **(C)**, TNF-α **(D)**, IL-8 **(E)**, and CCL3 **(F)** transcripts. The transcription level of each target was normalized to its own housekeeping gene level [glyceraldehyde 3-phosphate dehydrogenase (GAPDH)] and then compared with the untreated control (media only) to calculate the relative fold change. The bar charts show the data of three different donors. The results are presented as mean ± SD. The experiments were performed in triplicate with three independent experiments. One-way ANOVA followed by a Bonferroni multiple comparison test were used for statistical analysis: *p < 0.05, **p < 0.01, and ***p < 0.001. The treatment conditions were included media alone (Mock), only fMLP induction (fMLP), fMLP induction and AEBSF treatment (AEBSF+fMLP), fMLP induction and rTsSERP1 treatment (rTsSERP1+fMLP), fMLP induction and irrelevant control (rmDHFR) treatment (rmDHFR+fMLP).

Neutrophils were treated with rTsSERP1, AEBSF, or rmDHFR and then stimulated with fMLP. Treatment with rTsSERP1 significantly reduced the transcript levels of proinflammatory cytokines [IL-1β (donors 1 and 2; *p*<0.05 and donor 3; *p*<0.01) (**Figure 5A**), IL-6 (donor 1; *p*<0.001 and donors 2 and 3; *p*<0.05) (**Figure 5B**), IFN-γ (donor 1; *p*<0.05 and donors 2 and 3; *p*<0.01) (**Figure 5C**), and TNF-α (donor 1; *p*<0.001 and donors 2 and 3; *p*<0.01) (**Figure 5D**)] and chemokines [IL-8 (donor 1; *p*<0.001 and donors 2 and 3; *p*<0.01) (**Figure 5E**) and CCL3 (donors 2 and donor 3; *p*<0.05)(**Figure 5F**)] in fMLP-induced neutrophils. The outcomes of all three donors were consistent. Treatment of fMLP-stimulated neutrophils with rmDHFR did not suppress the transcript levels of proinflammatory cytokines and chemokines compared with fMLP-induced neutrophils alone (*p*>0.05) (**Figures 5A–F**). A positive inhibitor control (AEBSF) significantly downregulated the transcript levels of proinflammatory cytokines and chemokines from fMLP-induced neutrophils obtained from all three donors when compared with fMLP-induced neutrophils alone (*p*<0.05, *p*<0.01, and *p*<0.001, respectively). The level of transcription of neutrophil proinflammatory cytokines was not increased when neutrophils were treated with DMSO alone at the same concentration as that used to dissolve fMLP (**Supplementary Figure 6D**).

**Figure 6:**
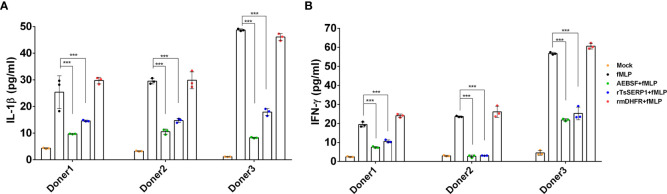
Inhibitory effect of rTsSERP1 on the production of proinflammatory cytokines released by human neutrophils. In addition to determining the transcription levels of cytokine and chemokine mRNAs in neutrophils, culture media were also collected for ELISA to analyze IL-1β **(A)** and IFN-γ **(B)** protein levels. ELISA results are reported as pg/ml. The bar charts show the data of three different donors, which are presented as the mean ± SD. The experiments were performed in triplicate with three independent experiments. One-way ANOVA followed by a Bonferroni multiple comparison test were used for statistical analysis: ***p < 0.001. The treatment conditions were included media alone (Mock), only fMLP induction (fMLP), fMLP induction and AEBSF treatment (AEBSF+fMLP), fMLP induction and rTsSERP1 treatment (rTsSERP1+fMLP), fMLP induction and irrelevant control (rmDHFR) treatment (rmDHFR+fMLP).

The protein levels of IL-1β and IFN-γ released from neutrophils were measured to confirm the qRT-PCR results. IL-1β and IFN-γ levels were significantly reduced when fMLP-induced neutrophils from all three donors were treated with rTsSERP1 (*p*<0.001). The levels of IL-1β and IFN-γ were reduced to 15–20 pg/ml (**Figure 6A**) and 3–26 pg/ml(**Figure 6B**), respectively, after rTsSERP treatment, whereas the levels of fMLP-induced neutrophils alone were higher 30–50 pg/ml and 20–57 pg/ml, respectively. There were no changes in IL-1β and IFN-γ production in fMLP-induced neutrophils treated with rmDHFR (*p*>0.05) (**Figures 6A, B**). AEBSF treatment of fMLP-induced neutrophils resulted in a significant reduction in IL-1β and IFN-γ levels (*p*<0.001). The incubation of neutrophils with rTsSERP1, rmDHFR, or AEBSF in the absence of fMLP-induction failed to increase the production of cytokines and chemokines at the transcript and protein levels (data not shown). According to the findings mentioned above, TsSERP1 reduced the amount of pro-inflammatory cytokines and chemokines produced by neutrophils, which could be mediated by NE inhibition.

## Discussion

Neutrophils, an essential component of the innate immune system, can kill *T. spiralis* NBL when they migrate through the blood (Bass and Szejda, 1979a; Bass and Szejda, 1979b). A high number of neutrophils was observed 3–17 days after *T. spiralis* infection and these gradually declined, possibly because of parasite immunomodulation (Wang et al., 2020). A *T. spiralis* 45 kDa glycoprotein (Tsgp45) was previously shown to suppress neutrophil migration, chemotaxis, and activation, resulting in anti-inflammatory effects (Bruschi et al., 2000). *T. spiralis* calreticulin (TsCRT) binds to C1q, inhibiting the classical complement pathway as well as C1q-associated neutrophil activation and NET formation (Shao et al., 2020). Apart from these molecules, TsSERPs, which were discovered in the ES of *T. spiralis*, were shown to inhibit neutrophil elastase and immunological functions of neutrophils, which was confirmed in this study.

Serpins are immunomodulators that mediate the regulatory protective effects of parasites against serine protease hydrolysis as well as being involved in various immune functions including apoptosis (Heutinck et al., 2010), coagulation (Loof et al., 2011), granzyme killing (de Koning et al., 2011; Niehaus et al., 2015), complement activation (Takahashi et al., 2007), and neutrophil functions (Pham, 2006). Several TsSERP isoforms have been identified and their potential as immunomodulators and vaccine candidates has been reported (Xu et al., 2021a).

TsSERP1, the first serpin isoform discovered from TsL1, inhibits trypsin (Nagano et al., 2001). We previously used rTsSERP1 for the immunodiagnosis of swine trichinosis and demonstrated that rTsSERP1 could diagnose late infection. In this study, we analyzed the inhibitory function of TsSERP1 against NSPs, which preferentially inhibited hNE and partially inhibited hCG but not hPR3. A serpin of the human hookworm *Ancylostoma duodenale* (AduTIL-1) suppressed digestive enzymes and NE (Jin et al., 2011). Bm-spn-2, a *Brugia malayi* serpin, suppressed CG and NE specifically (Zang et al., 1999). The impact of helminth serpins on neutrophil functions, which was originally examined using TsSERP1 in this study, has yet to be determined elsewhere.

The specificity and function of serpins that inhibit serine proteases have been intensively studied, and they depend on two regions of the reactive central loop (RCL) at the P1 residue and a highly-conserved hinge region at residues P15–P9 (Marijanovic et al., 2019). TsSERP1 and alpha-1 antitrypsin (A1AT) have relatively similar P1 and P15–P9 regions, except for P10, which is alanine (A) for TsSERP1 and glycine (G) for A1AT (**Supplementary Figure 7**). A1AT can inhibit a diverse variety of NSPs, especially NE (Marijanovic et al., 2019), whereas TsSERP1 specifically inhibits NE. Differences in substrate specificity between TsSERP1 and A1AT might be related to variances in the P8–P3 or P2’–P3’ regions or the insertion of a MYES motif at P4’–P8’ in TsSERP1. This should be investigated further *via* mutagenesis studies.

Neutrophils are a type of innate immune cell that serves as a first line of defense against infections. The secretion of NSPs such as NE, PR3, and CG is critical in bacterial killing and facilitating neutrophil immune responses (Korkmaz et al., 2010). NE is a major enzyme that has important roles in neutrophil activation as well as host tissue damage (Gramegna et al., 2017). Silvelestat, a small molecule inhibitor of NE, reduced neutrophil inflammation, NET production, and NET-mediated tissue injury (Hagiwara et al., 2008; Okeke et al., 2020). Mice lacking NE and CG were demonstrated to be more sensitive to bacterial and fungal infection (Tkalcevic et al., 2000). These findings suggest that TsSERP1 secreted from *T. spiralis*, which inhibits NE preferentially, might be an essential immune evasion mechanism against neutrophils.

In this study, rTsSERP1 suppressed NE activity in the culture media of fMLP-induced neutrophils, as well as phagocytosis. Phagocytosis is the ability of neutrophils to ingest and destroy invading microorganisms, particularly bacteria and fungus, which is critical for the health of the host (Kobayashi et al., 2018). Helminths are large pathogens and therefore are difficult for neutrophils to phagocytose. Furthermore, the intracellular infection of *T. spiralis* in host muscle and nurse cell formation can protect parasites from host immune cells including neutrophils (Wu et al., 2008). NET formation (NETosis) is an important process for defense against parasitic helminths by trapping them in NETs, which restricts parasite mobilization (Díaz-Godínez and Carrero, 2019; Hermosilla et al., 2014). Human neutrophils were stimulated to produce NETs by *Strongyloides stercoralis* larvae *in vitro*, which aided larval death (Bonne-Année et al., 2014). *Haemonchus contortas* third-stage larvae (L3) activated NET formations, trapping and killing the larvae (Muñoz-Caro et al., 2015). In NETosis, NE is a major protease in NETosis that translocates from azurophilic granules into the nucleus by reactive oxygen species (ROS) and myeloperoxidase (MPO), and which partially cleaves histone to enhance chromatin condensation (Metzler et al., 2014). NET production could be inhibited by NE inhibitors as SLPI and serpin B1 (Majewski et al., 2016). Furthermore, compared with wild-type mice, serpinB1 knockout mice had an increased release of NETs in PMA-activated neutrophils (Farley et al., 2012). These findings support our findings that rTsSERP1 prevents NET formation in PMA-activated neutrophils. However, the exact mechanism by which TsSERP1 modulates NETosis during helminthic infection or unrelated inflammatory disorders is unknown.

Apart from their primary defense against infections, neutrophils also provide an immunomodulatory role, which involves their NSPs processing cytokines and chemokines directly and indirectly to initiate inflammatory responses. The inhibition of NE by NE inhibitors decreased the production of proinflammatory cytokines in inflammation-stimulated neutrophils and monocytes (Haga and Ogawa, 1997). In NE-deficient mice, the transcript and protein levels of the proinflammatory cytokines TNF-α, MIP-2, and IL-6, in the lungs were decreased, which was associated with increased mortality from *P. aeruginosa* infection. Two recombinant *T. spiralis* serpins, rTsKaSPI and rTsAdSPI, ameliorated the severity of TNBS-induced colitis, which was related to the decreased expression of IFN-γ, a proinflammatory cytokine that interacts with multiple signaling pathways of the innate and adaptive immune responses (Xu et al., 2018). *Schistosoma mansoni* Kunitz type protease inhibitor (*Sm*KI-1) inhibited NE, which impaired neutrophil function and inflammation. Treatment of various murine models of inflammatory diseases, including acetaminophen (APAP)-mediated liver damage, gout arthritis, and carrageenan-induced plural effusion with *Sm*KI-1 impaired neutrophil migration, NE activity, and IL-1β secretion, which ameliorated clinical manifestations (Morais et al., 2018). In fMLP-primed neutrophils, rTsSERP1 significantly reduced the production of proinflammatory cytokines (IL-1β, IL-6, IFN-γ, and TNF-α) and chemokines (IL-8 and CCL3). These findings suggest the therapeutic potential of rTsSERP1 for the treatment of inflammatory disorders.

**Figure 7:**
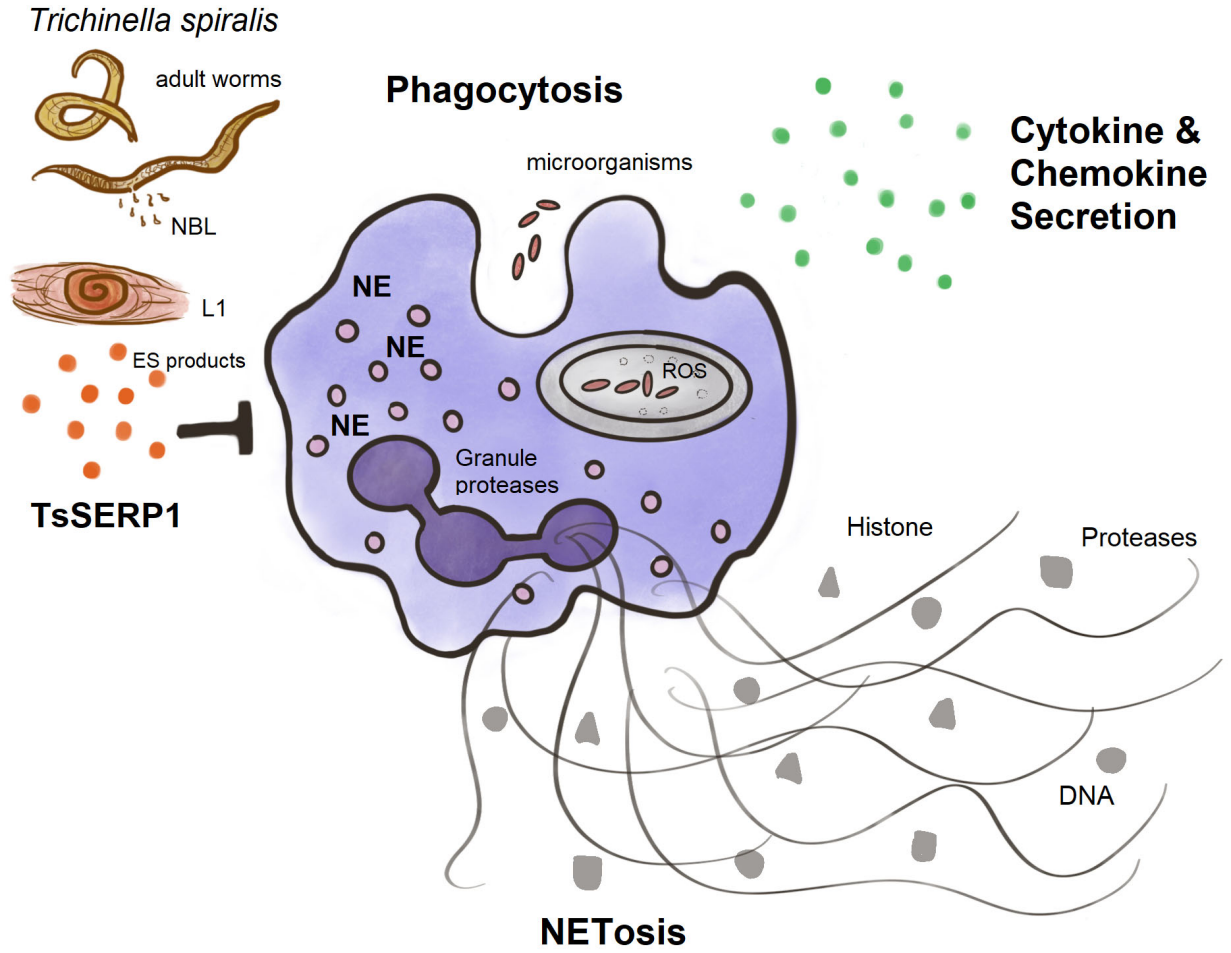
A diagram summarizing the main findings of this study. TsSERP1, presented at different developmental stages of *T. spiralis* and its excretory-secretory (ES) products, inhibited the hNE activity of human neutrophils. The inhibitory effect of TsSERP1 on hNE influences neutrophil functions by impairing phagocytosis, NETosis, and the production of proinflammatory cytokines and chemokines.

Finally, TsSERP1 suppressed NE and impaired neutrophil activities including phagocytosis, NETosis, and the production of proinflammatory cytokines and chemokines (**Figure 7**). Our future study will focus on providing insights into the mechanism related to the *ex vivo* and *in vivo* regulation of neutrophils by TsSERP1. In addition, the therapeutic potential of rTsSERP1 in a mouse model of inflammatory disease must be studied. Understanding the immunomodulatory functions of neutrophils might lead to the development of novel therapeutics for acute and chronic inflammatory disorders.

## Data availability statement

The original contributions presented in the study are included in the article/**Supplementary Material**, further inquiries can be directed to the corresponding author/s.

## Ethics statement

The studies involving human participants were reviewed and approved by The Human Research Ethics Committee of the Faculty of Tropical Medicine, Mahidol University, Bangkok, Thailand (MUTM 2021-013-01). Written informed consent for participation was required for this study in accordance with the national legislation and the institutional requirements.

## Author contributions

PK performed all work, designed experiments, analyzed data and drafted the manuscript. OR, OP, and PM provided reagents and designed the experiments. PA conceived and designed the experiments, analyzed data and drafted the manuscript. All authors read the manuscript and approved the submitted version.

## Funding

This research project was supported by Mahidol University under the New Discovery and Frontier Research grant Fiscal Year 2021 through Poom Adisakwattana (NDFR 03/2564) and the Thailand Research Fund under TRF Research Career Development Grant through Poom Adisakwattana (RSA6180072). This study was also supported by ICTM Grants from the Faculty of Tropical Medicine, Mahidol University. The funders had no role in study design, data collection and analysis, decision to publish, or preparation of the manuscript.

## Acknowledgments

We are grateful to Mahidol University’s Faculty of Tropical Medicine for providing all facilities and services. We are also grateful to the Central Equipment Unit of Mahidol University’s Faculty of Tropical Medicine for providing us with all of the necessary tools. We thank J. Ludovic Croxford, PhD, from Edanz (www.edanz.com/ac) for editing a draft of this manuscript.

## Conflict of interest

The authors declare that the research was conducted in the absence of any commercial or financial relationships that could be construed as a potential conflict of interest.

## Publisher’s note

All claims expressed in this article are solely those of the authors and do not necessarily represent those of their affiliated organizations, or those of the publisher, the editors and the reviewers. Any product that may be evaluated in this article, or claim that may be made by its manufacturer, is not guaranteed or endorsed by the publisher.
