# Supplementary material for: Serine protease inhibitor derived from Trichinella spiralis (TsSERP) inhibits neutrophil elastase and impairs human neutrophil functions
Source: Front Cell Infect Microbiol. 2022 Oct 25;12:919835. doi: 10.3389/fcimb.2022.919835 (PMC9640929; doi:10.3389/fcimb.2022.919835)
Supplement: Supplementary file 1 [file DataSheet_1.docx]

Supplementary Material

**Supplementary Table 1** The gene specific primers for SYBR green qRT-PCR

| **Gene name** | **Function** | **Primer sequences (5’ 🡪 3’)** | **Accession no.** | **Length (bp)** |
| --- | --- | --- | --- | --- |
| IL-1β | Interleukin-1β | Fw: 5’-CCTACTCACTTAAAGCCCGCCTG-3’  Rw: 5’-CACTGCTACTTCTTGCCCCCTTT-3’ | NM_000576 | 174 |
| IL-6 | Interleukin-6 | Fw: 5’-ACTCACCTCTTCAGAACGAATTG-3’  Rw: 5’-CCATCTTTGGAAGGTTCAGGTTG-3’ | NM_000600.5 | 149 |
| IFN-γ | Interferon-γ | Fw: 5’-CAACTAGGCAGCCAACCTAAGCA-3’  Rw:5’-GCAGGCATATTTTCAAACCGGCA-3’ | NM_000619.3 | 123 |
| TNF-α | Tumor necrotic factor-α | Fw:5’-TGTACTCCTCACCCACACCATCA-3’  Rw:5’-TAGATGGGCTCATACCAGGGCTT-3’ | MH180383.1 | 138 |
| IL-8 | Interleukin-8 | Fw:5’-ACTGAGAGTGATTGAGAGTGGAC-3’  Rw:5’-AACCCTCTGCACCCAGTTTTC-3’ | AK311874.1 | 383 |
| CCL3 | Chemokine ligand-3 | Fw:5’-TCAGACTTCAGAAGGACACGG-3’  Rw:5’- CTGCATGATTCTGAGCAGGTG-3’ | EF064769.1 | 100 |
| GAPDH | House keeping | Fw:5’-GCATCCTGGGCTACACTGAG-3’  Rw:5’-TGCTGTAGCCAAATTCGTTG-3’ | NM_002046 | 150 |


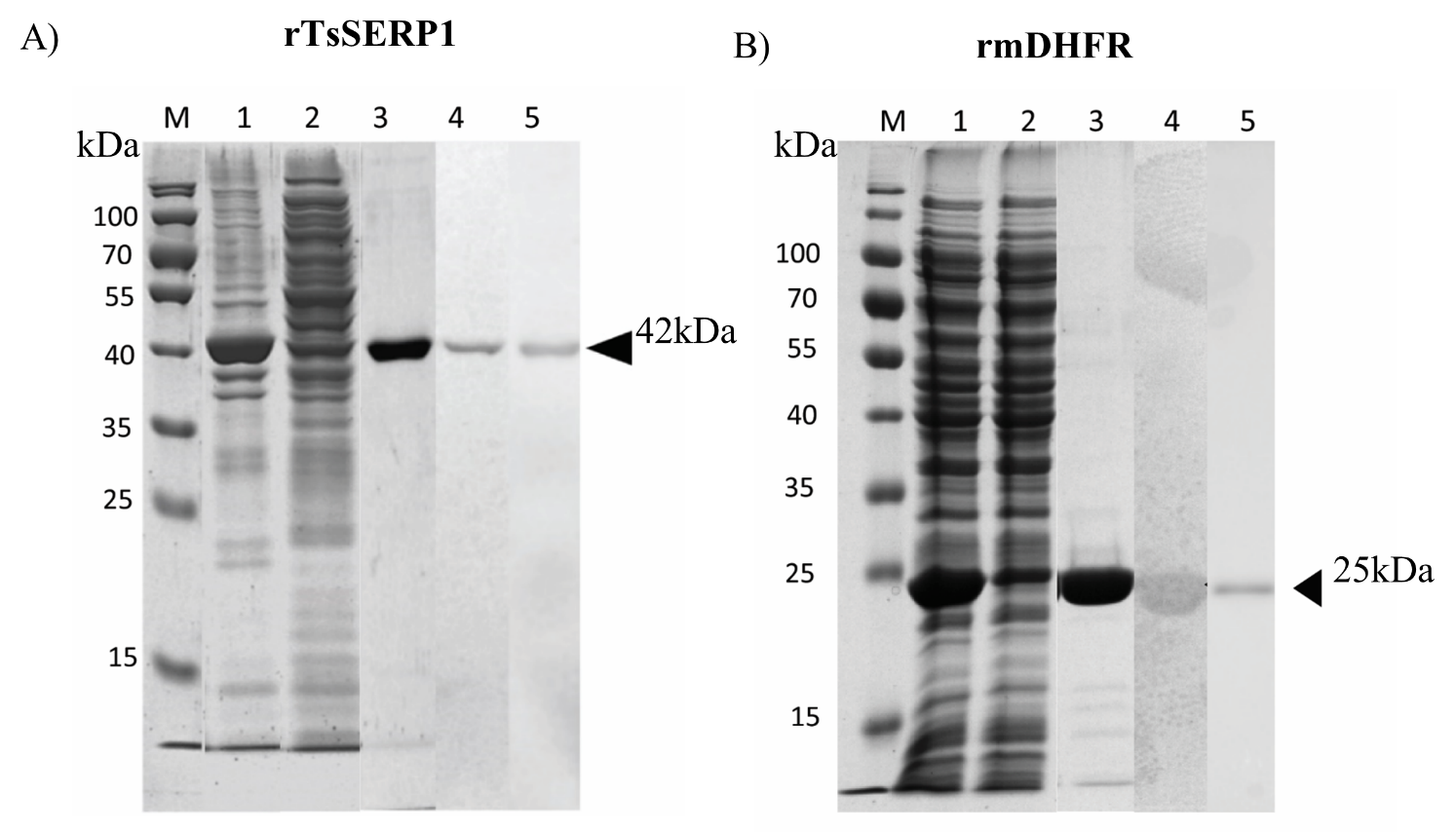


**Supplementary Figure 1** The expression and purification of rTsSERP1 (**A**) and rmDHFR (**B**). rTsSERP1 and rmDHFR were expressed in *E. coli* by induction of 0.5 mM isopropyl-β-D-thiogalactopyranoside (IPTG; Thermo Fisher Scientific) at 37 °C for 3 h. The fusion protein was obtained after centrifugation at 12,000 ×g for 30 min at 4°C, followed by purification using CO2+ affinity chromatography (Clontech Laboratories, Inc., Mountain View, CA) under the native buffer conditions (50 mM NaH_2_PO_4_, 300 mM NaCl, pH 6.3 containing an imidazole at 250 mM). The expression and purification of rTsSERP and rmDHFR were analyzed on a 12% SDS-PAGE and western blot using an anti-His tag antibody (BioLegend, San Diego, CA). The endotoxin contaminated protein solution was removed by a phase separation technique using Triton-X114 (Sigma-Aldrich). The residual endotoxin of rTsSERP (0.054 EU/ml) and rmDHFR (0.06 EU/ml) was assessed by Pierce^TM^ LAL Chromogenic Endotoxin Quantitation Kit (Thermo Fisher Scientific). M; PageRuler Prestained Protein Ladder (Thermo Scientific), Lane 1; induced IPTG, soluble protein, Lane 2; induced IPTG, insoluble protein, Lane 3; the purified soluble protein, Land 4; western blot analysis of the purified protein, Lane 5; western blot analysis of endotoxin removed protein.


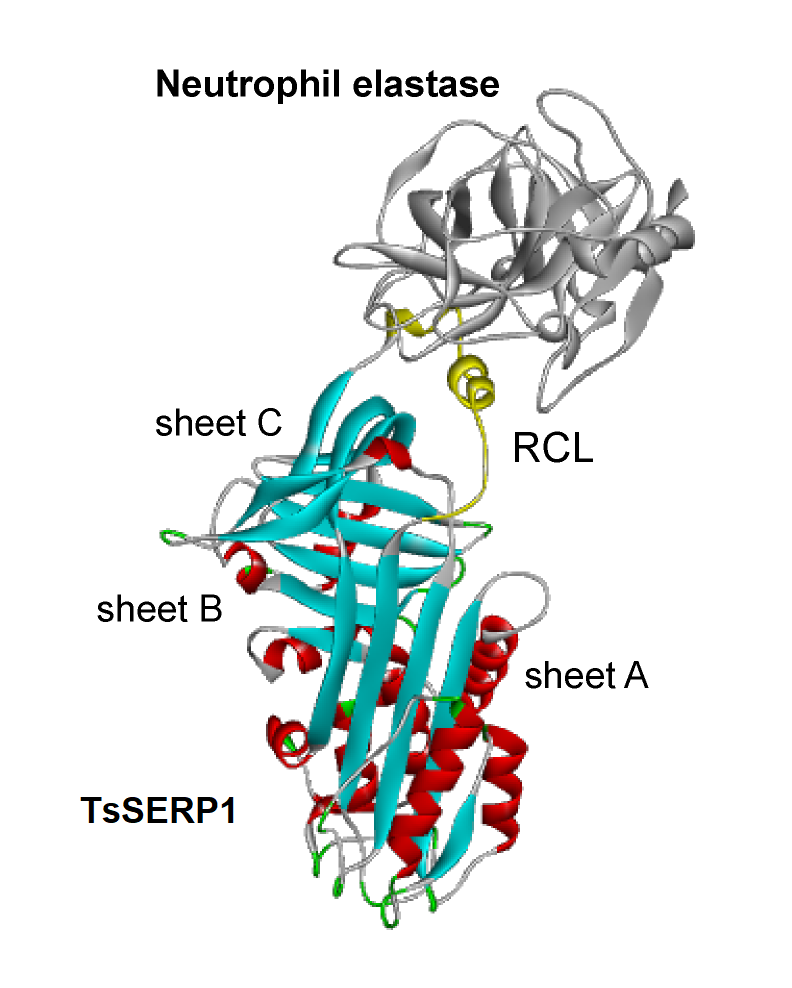


**Supplementary Figure 2** Docking model of TsSERP1- human neutrophil elastase (hNE) interaction. The 3D structure of TsSERP1 was constructed by Swissmodel^59^ using conserpin (PDB code: [5cdx.1.A](file:///C:\Users\hp01\AppData\Local\Temp\Temp1_TsSERP_03_2021-08-15.zip\models\03\templates\5cdx.1.A.pdb.gz)) as the homologous protein template sharing 42.49% sequence identity. The structure of hNE was obtained from protein data bank (PDB code: 3q76). The interaction between TsSERP1 and hNE (PDB code: 3q76) was analysed using ZDOCK server^60^. The TSSERP1 structure was labelled in this view with α-helices (red), β-strands (blue), irregular coils (green) and reactive central loop (yellow), whereas hNE structure was labelled in gray.


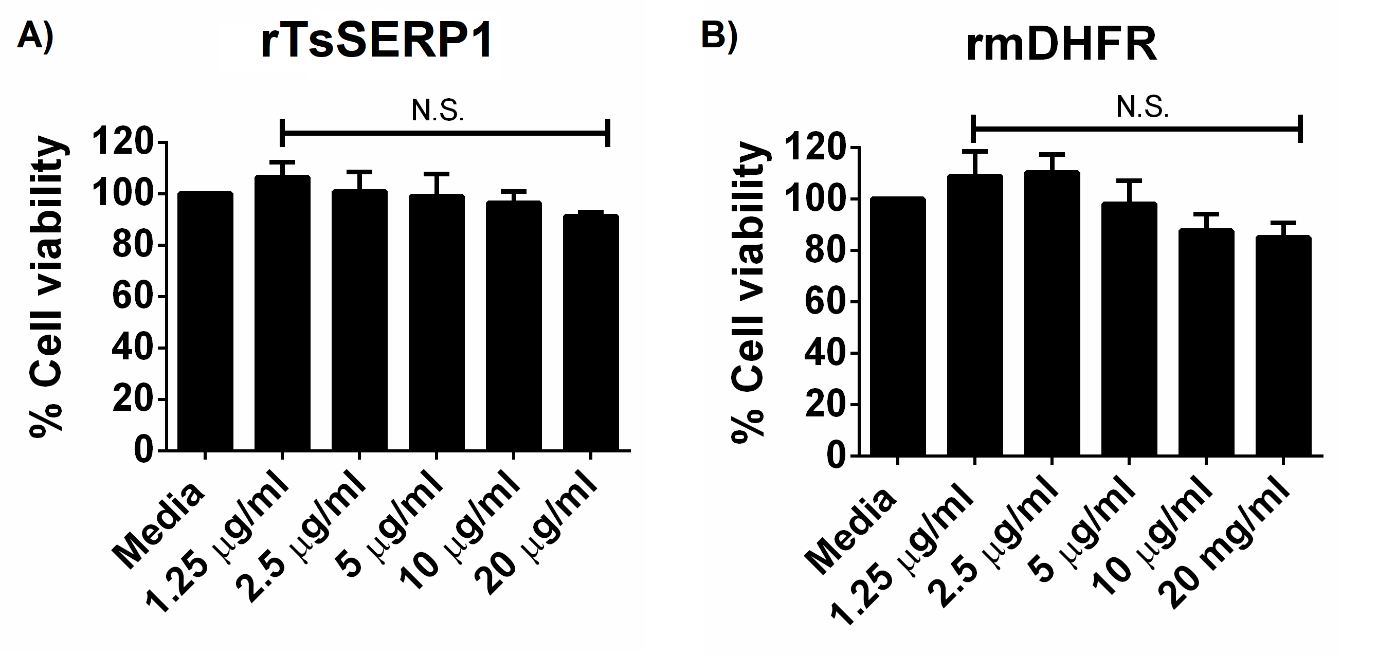


**Supplementary Figure 3** Effect of rTsCstN and rmDHFR on cell viability of human neutrophils using MTT assay. The cells treated with medium alone were used as negative control. The protein concentrations of rTsSERP1 or rmDHFR were in the range between 1.25-20 μg/ml. The data presented as percentage cell viability in mean ± SD of triplicates. N.S., not significant (p>0.05) represents low cytotoxicity.


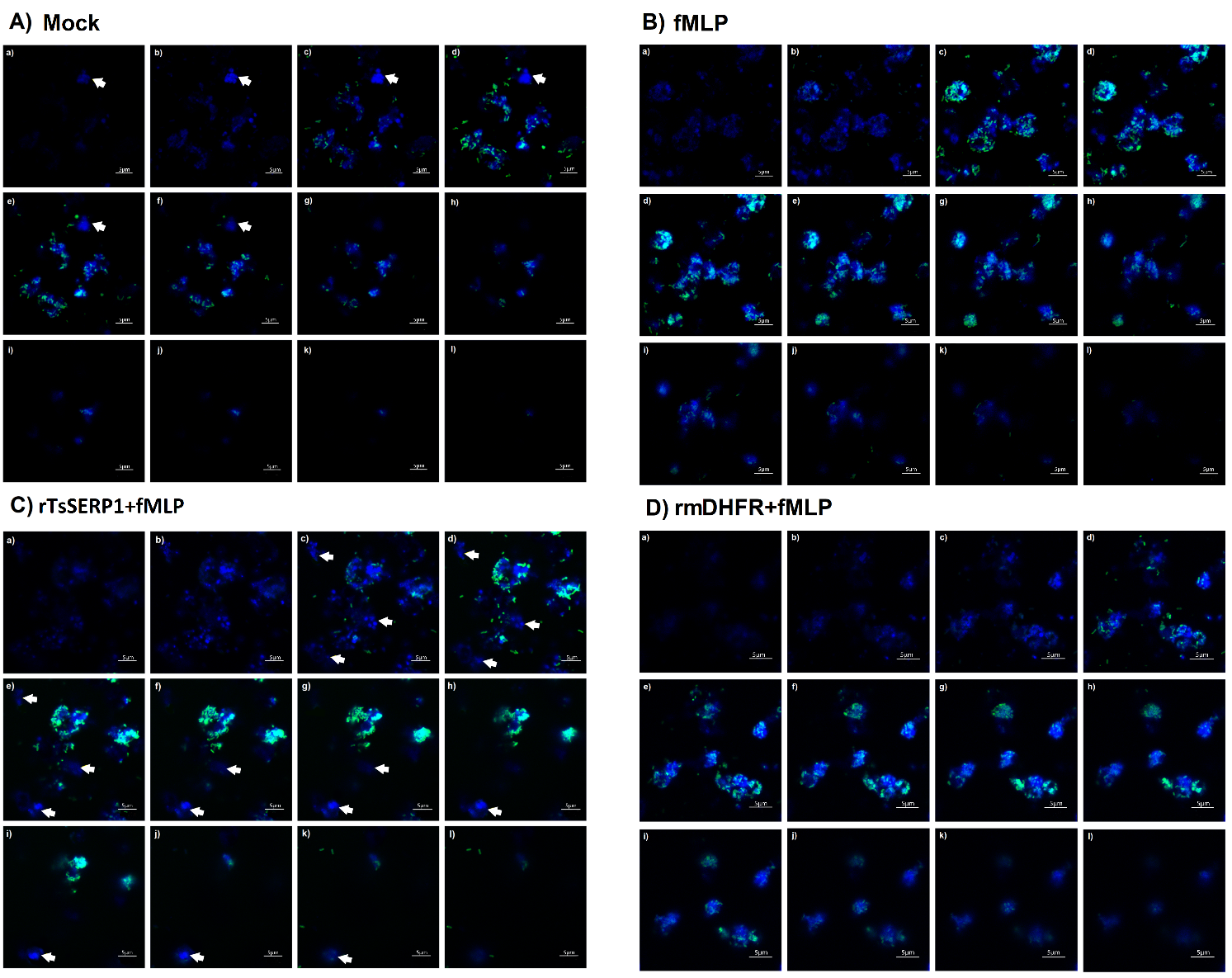


**Supplementary Figure 4** The Z-stack analysis to confirm the neutrophil phagocytosis of FITC labelled *E. coli* BioParticle (green) when treatment with different conditions including media alone (A: Mock), only priming with fMLP (B: fMLP), fMLP induction and rTsSERP1 treatment (C: rTsSERP1+fMLP), fMLP induction and rmDHFR treatment (D: rmDHFR+fMLP). The nucleus was counterstained with Hoechst 33342 (Blue). Examples of non-phagocytotic neutrophils are indicated by white arrows.


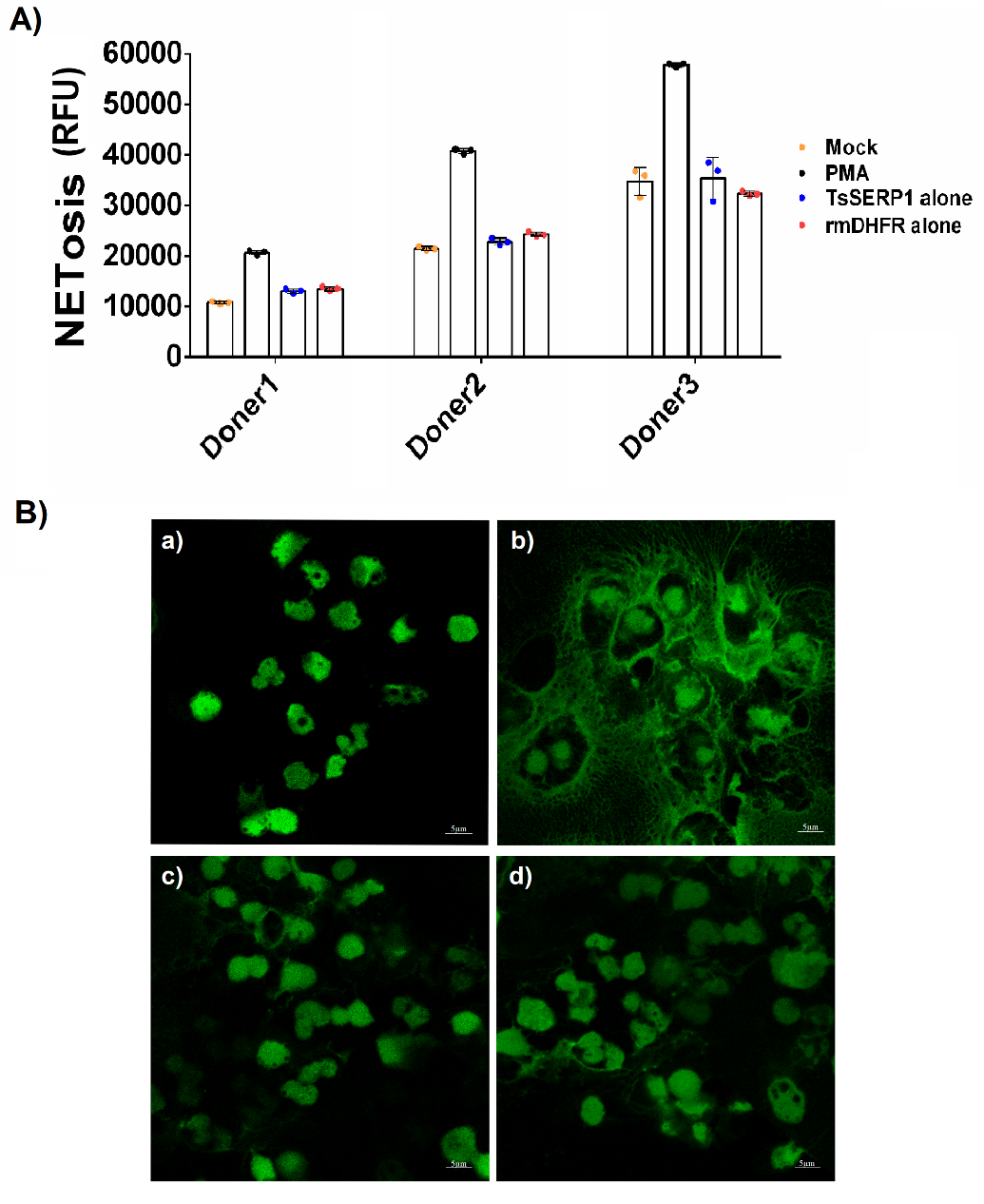


**Supplementary Figure 5** Neutrophil did not induced NETs in the only media or treatment with rTsSERP1 or rmDHFR alone when compared to PMA stimulation. The NETosis was analyzed by the quantitative fluorescence intensity (**A**) and fluorescent images (**B**) with different treatment conditions including media alone (Mock) (a), PMA induction alone (PMA) (b), rTsSERP1 treatment (rTsSERP1 alone) (c), and treatment with an irrelevant control (rmDHFR) (rmDHFR alone) (d) The bar charts show the data of three different donors, which are presented as the mean ± SD. The experiments were performed in triplicate with three independent experiments.


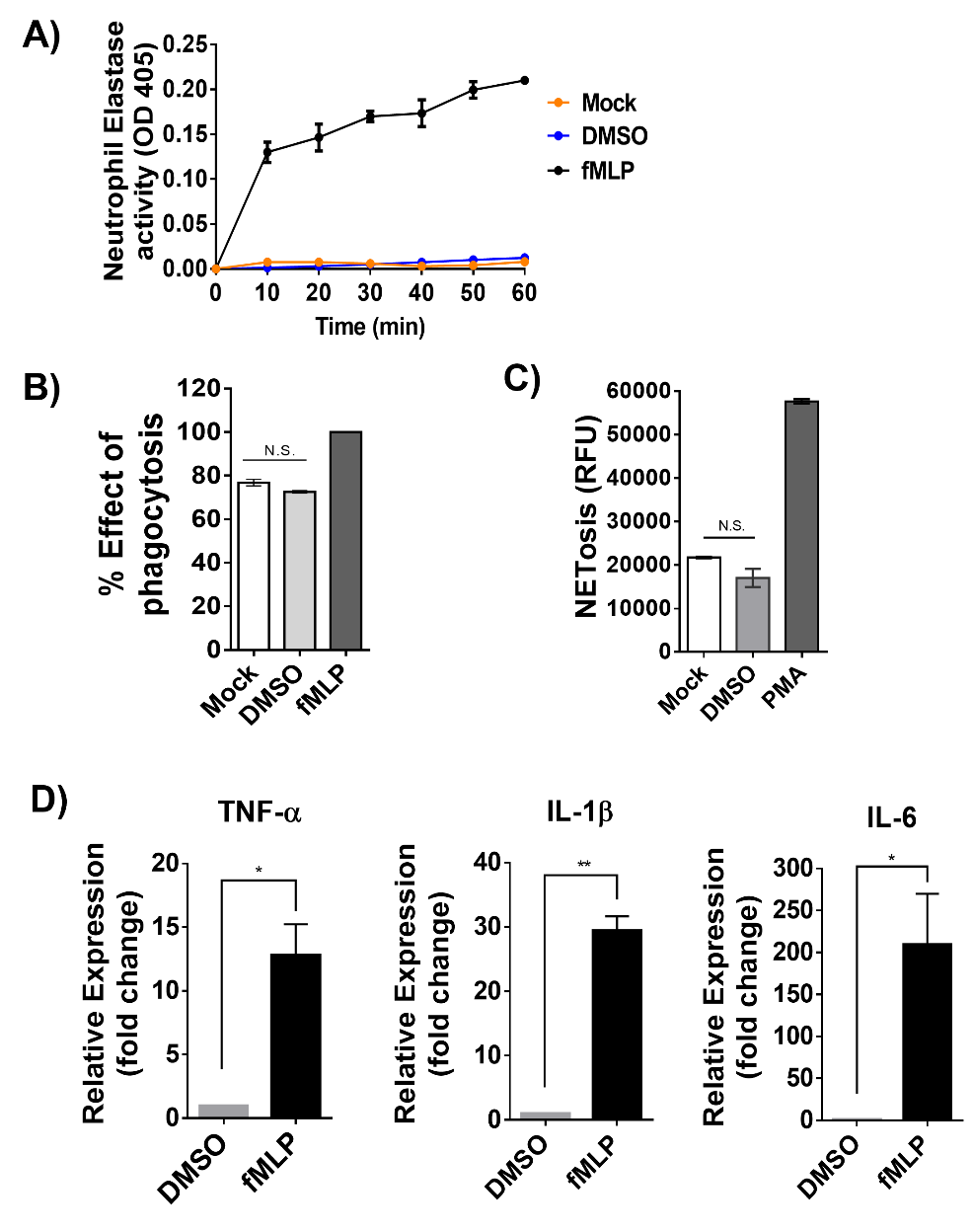


**Supplementary Figure 6** Neutrophils treated with DMSO alone had no effect on hNE activity (**A**), phagocytosis (**B**), NETosis (**C**), and transcription level of proinflammatory cytokines (**D**). The same amount of DMSO used to dissolve either 100 nM fMLP or 50 nM PMA was treated with human neutrophils, and the experiments were performed in triplicates to detect hNE activity, the percentage effect of phagocytosis, NETosis, and transcription level of proinflammatory cytokines (TNF-α, IL-1β and IL-6). The line and bar charts showed the mean ± SD. Statistical analysis showed N.S. was non-significant results, **p* ˂ 0.05 and ***p* ˂ 0.01, respectively.


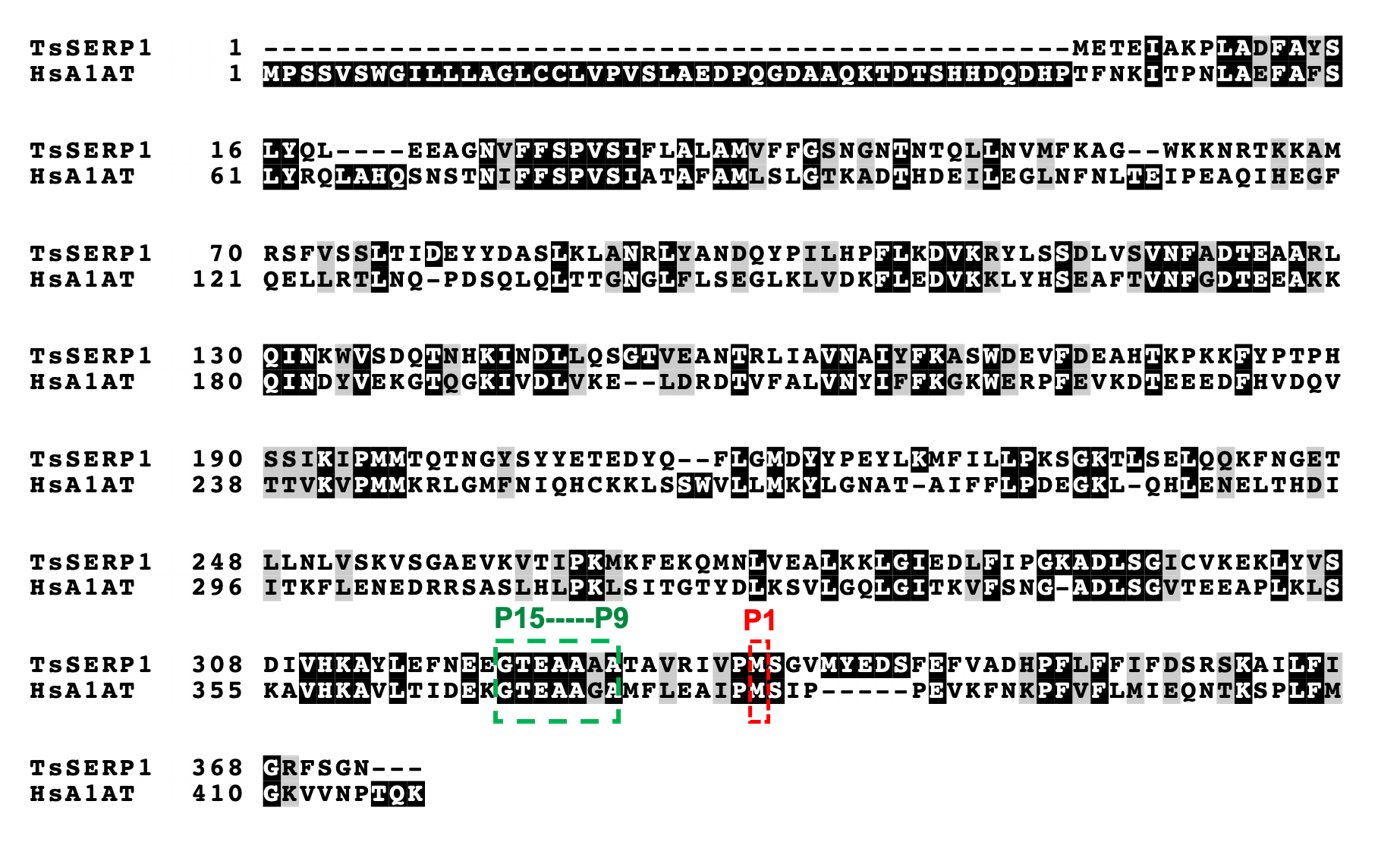


**Supplementary Figure 7** Multiple alignments of TsSERP1 and alpha-1 antitrypsin (A1AT). Amino acid sequences were aligned and displayed using Clustal Omega and BoxShade server (https://embnet.vital-it.ch/software/BOX_form.html), respectively. The similarity of conserved domain between TsSERP1 and A1AT was presented in P1 (red box) and P15-P9 regions (green box).
